# RETRACTED ARTICLE: DynaGraph: interpretable dynamic graph learning for temporal electronic health records

**DOI:** 10.1038/s41746-025-02328-0

**Published:** 2026-01-16

**Authors:** Munib Mesinovic, Soheila Molaei, Peter Watkinson, Tingting Zhu

**Affiliations:** 1 Department of Engineering Science, University of Oxford, Oxford, UK; 2 Nuffield Department of Clinical Neurosciences, University of Oxford, Oxford, UK

**Keywords:** Computational biology and bioinformatics, Diseases, Health care, Mathematics and computing, Medical research

The Editor-in-Chief has retracted this article.

For this article, several concerns have been raised regarding irregularities in the content and in some of the cited references. Issues include incorrect statements, inconsistencies in reported time intervals, incorrect equations or missing notations, irregularities in provided code and the presence of non-existing references. The authors have provided an explanation for the raised concerns, and have acknowledged some of the inconsistencies in content; however, their explanation does not appear to be satisfactory and does not resolve the raised concerns.

Therefore, the Editor-in-Chief has lost confidence in the content and data presented in this article.

Peter Watkinson stated on behalf of Tingtin Zhu that they agree with this retraction. None of the remaining authors has responded to any correspondence regarding this retraction.

The online version of this article contains the full text of the retracted article as Supplementary Information.

## Supplementary information

Former article version

