# Supplementary material for: RETRACTED ARTICLE: DynaGraph: interpretable dynamic graph learning for temporal electronic health records
Source: NPJ Digit Med. 2026 Jan 16;9:216. doi: 10.1038/s41746-025-02328-0 (PMC12992538; doi:10.1038/s41746-025-02328-0)
Supplement: Supplementary file 1 — Former article version [file 41746_2025_2328_MOESM1_ESM.pdf]

npj Digital Medicine

https://doi.org/10.1038/s41746-025-02328-0

Article in Press

RETRACTED ARTICLE: DynaGraph: interpretable dynamic graph learning for temporal electronic health records

Received: 31 July 2025

Accepted: 26 December 2025

[redacted]

Cite this article as: Mesinovic, M., Molaei, S., Watkinson, P. *et al.* RETRACTED ARTICLE: DynaGraph: interpretable dynamic graph learning for temporal electronic health records. *npj Digit. Med.* (2026). https://doi.org/10.1038/s41746-025-02328-0

Munib Mesinovic, Soheila Molaei, Peter Watkinson & Tingting Zhu

We are providing an unedited version of this manuscript to give early access to its findings. Before final publication, the manuscript will undergo further editing. Please note there may be errors present which affect the content, and all legal disclaimers apply.

If this paper is publishing under a Transparent Peer Review model then Peer Review reports will publish with the final article.

RETRACTED ARTICLE

© The Author(s) 2026. **Open Access** This article is licensed under a Creative Commons Attribution 4.0 International License, which permits use, sharing, adaptation, distribution and reproduction in any medium or format, as long as you give appropriate credit to the original author(s) and the source, provide a link to the Creative Commons licence, and indicate if changes were made. The images or other third party material in this article are included in the article's Creative Commons licence, unless indicated otherwise in a credit line to the material. If material is not included in the article's Creative Commons licence and your intended use is not permitted by statutory regulation or exceeds the permitted use, you will need to obtain permission directly from the copyright holder. To view a copy of this licence, visit http://creativecommons.org/licenses/by/4.0/.

DynaGraph: Interpretable Dynamic Graph Learning for Temporal Electronic Health Records

Munib Mesinovic[1*], Soheila Molaei[1], Peter Watkinson[2], Tingting Zhu[1]

[1*]Department of Engineering Science, University of Oxford, Oxford, UK.
[2]Nuffield Department of Clinical Neurosciences, University of Oxford, Oxford, UK.

*Corresponding author(s). E-mail(s): munib.mesinovic@eng.ox.ac.uk;
Contributing authors: soheila.molaei@eng.ox.ac.uk; peter.watkinson@ndcn.ox.ac.uk; tingting.zhu@eng.ox.ac.uk;

RETRACTED ARTICLE

Abstract

Electronic health records (EHRs) capture evolving physiological processes, yet most machine learning models impose static or sequential assumptions that flatten their temporal and relational complexity. We introduce DynaGraph, a dynamic and interpretable graph learning framework that constructs evolving spatio-temporal graphs from multivariate clinical time-series. Unlike previous methods, DynaGraph learns the structure of relationships between different clinical variables over time without predefined graphs, integrates sequential embeddings with contrastive graph augmentation, and incorporates a pseudo-attention mechanism to reveal temporally resolved risk factors. Trained end-to-end with a novel multi-loss objective that combines focal, structural, and contrastive components, DynaGraph addresses two pervasive challenges in real-world clinical modelling: class imbalance and temporal instability. We evaluated DynaGraph on four large-scale EHR datasets totalling 40,856 patients: MIMIC-III (17,279 ICU admissions), eICU (1,433 cardiac ICU patients), HiRID-ICU (33,000 patients), and EHRSHOT (2,378 primary care patients). DynaGraph consistently outperforms 14 state-of-the-art baselines, achieving 6-8% relative improvements in area under the precision-recall curve (AUPRC) and significant gains in sensitivity (12-22% over leading methods). Beyond predictive performance, DynaGraph offers time-specific interpretability aligned with clinical reasoning, providing gradient-based feature importance scores at 3-hour intervals that identify which physiological relationships drive predictions. This framework explicitly models temporal attribution of risk factors across patient trajectories in a millisecond inference time.

Keywords: graphs, healthcare, interpretability, machine learning, time-series

Introduction

Clinical deterioration rarely follows linear trajectories. Instead, it unfolds through cascading interactions between organ systems that evolve over distinct temporal phases. Consider a patient developing septic shock: initial infection triggers inflammatory cascades (hours 0-12), followed by cardiovascular instability and renal hypoperfusion (hours 12-24), culminating in multi-organ dysfunction where hepatic, coagulation, and neurological systems become interdependent (beyond 24 hours). This exemplifies a fundamental challenge in clinical prediction where the relationships between physiological variables are neither static nor independent but dynamically restructure as patients transition between clinical states.

Electronic health records capture these evolving physiological processes through multivariate time-series including vital signs, laboratory values, and clinical observations. These data fuel a growing ecosystem of machine learning models for early diagnosis, outcome prediction, and risk stratification across clinical settings [1–3]. Standard machine learning methods fail to accommodate the mixed data types, variable sampling frequencies, and large feature sets characteristic of real-world EHRs. Many models also disregard the structure of relationships between different clinical variables and offer limited transparency [4].

Recent advances in deep learning have led to increasingly powerful models for temporal EHR data, including extensions on long short-term memory (LSTM) networks, gated recurrent units (GRUs), and temporal convolutional networks (TCNs) [5–9]. Transformer variants such as Medformer, BiT-MAC, RETAIN, and PatchTST have improved long-range temporal modelling while offering partial interpretability [10–14]. These architectures, however, largely treat features independently and operate on fixed or sequential assumptions. For instance, they cannot capture how the coupling between creatinine and blood pressure strengthens during cardiorenal syndrome but weakens during recovery, missing the evolving relationships between physiological variables over time.

Graph-based models offer a powerful alternative by explicitly modelling structure. Graph neural networks (GNNs) can capture inter-variable interactions and perform robust reasoning even with missing data [15–17]. Classical approaches like graph convolutional networks (GCNs), graph attention networks (GATs), temporal graph attention (TGAT), and GraphSAGE enable neighbourhood-aware learning [18–21]. In traditional implementations, nodes might represent laboratory values while edges encode known physiological relationships—sodium connects to potassium through electrolyte balance, creatinine links to urea through renal function. More recent spatio-temporal GNNs extend these ideas to model temporal evolution via recurrent or convolutional updates [22–24]. Yet most prior work relies on fixed or hand-designed graphs derived from medical ontologies or correlation thresholds, assuming that the

RETRACTED ARTICLE

strength of connection between, for instance, heart rate and lactate remains constant from admission to discharge. This contradicts extensive clinical evidence showing that organ crosstalk intensifies during critical illness and restructures during recovery phases. Furthermore, these approaches lack interpretable mechanisms to reveal which relationships drive predictions at specific time points, thereby limiting both flexibility and transparency in dynamic clinical settings [25–27].

In this work, we introduce DynaGraph, a fully dynamic and interpretable graph learning framework designed for multivariate clinical time-series. DynaGraph constructs spatio-temporal graphs end-to-end from raw EHR signals without predefined adjacency matrices, integrating sequential embeddings with dynamic adjacency learning and a pseudo-attention mechanism to reveal time-resolved feature importances. By learning structure jointly with node representations, DynaGraph captures the changing topology of clinical variables and thereby discovers how relationships between features shift across a patient's journey rather than imposing predetermined patterns.

Our primary contribution is enabling dynamic structure learning: the model learns which physiological variables connect and how these connections evolve over time, without requiring predefined graphs from medical ontologies. This addresses the fundamental limitation that existing graph-based clinical models cannot adapt their structure to patient-specific trajectories. To ensure robust learning in realistic clinical settings, we incorporate a multi-loss training objective with contrastive graph augmentation, focal loss for severe class imbalance (with outcomes ranging from 2-38% prevalence), and structural regularisation for temporal stability. While handling class imbalance is essential for clinical deployment, it represents a necessary implementation consideration rather than our core methodological innovation.

We evaluate DynaGraph on four real-world EHR datasets spanning intensive and primary care domains. Across all tasks, it outperforms leading time-series and graph-based models, achieving 6-8% relative improvements in area under the precision-recall curve (AUPRC), particularly critical given severe class imbalance. More importantly, DynaGraph highlights clinically aligned and temporally specific risk factors: early emphasis on demographic factors transitioning to organ-specific markers, elevated renal-cardiovascular coupling preceding adverse events, and progressive decoupling of inflammatory markers during recovery. The model achieves 6-8% AUPRC improvements while providing time-resolved feature importance scores through gradient-based attribution. In doing so, DynaGraph bridges a critical gap between relational modelling, temporal dynamics, and interpretability in healthcare AI. It performs consistently across four datasets (AUROC 0.802-0.881) and reveals which feature pairs (e.g., creatinine-urea coupling ¿ 0.8) indicate clinical deterioration at specific time points.

Results

Study Design

We evaluated our proposed framework (Figure 1) across four large-scale EHR datasets encompassing 40,856 patients from intensive and primary care settings. The MIMIC-III cohort comprised 17,279 ICU admissions (mean age 74.1±13.4 years, 57.1% male,

RETRACTED ARTICLE

71.1% Caucasian) with 10 binary outcomes, including mortality (9.65% prevalence) and organ failures (2.19-32.60% prevalence). The eICU dataset included 1,433 cardiac ICU patients (mean age 67.2±12.4 years, 64.5% male) monitored for post-myocardial infarction complications (16.0% developed at least one complication). HiRID-ICU contained 33,000 ICU admissions with 5-minute resolution data, while EHRSHOT represented 2,378 primary care patients tracked for the development of chronic diseases. All cohorts were split 80:10:10 for training, validation, and testing with patient-level stratification.

Input features comprised variables including static demographics (age, sex) and hourly time-series measurements: vital signs (heart rate, blood pressure, temperature, respiratory rate, oxygen saturation), basic metabolic panel (sodium, potassium, chloride, bicarbonate, glucose, BUN, creatinine), complete blood count (haemoglobin, haematocrit, white cells, platelets), hepatic markers (bilirubin, alkaline phosphatase, AST, ALT), and coagulation studies (PT, PTT, INR). Missing values were forward-filled up to 6 hours, then imputed using patient-specific medians.

DynaGraph differs fundamentally from existing approaches by learning graph structures dynamically from data rather than using predefined medical ontologies. The framework segments 24-hour patient trajectories into six 4-hour windows. For each window, it constructs a graph where nodes represent the clinical features and edges capture learned relationships between them. Unlike previous dynamic graph models, which require predetermined adjacency matrices from medical knowledge bases, or LSTM/Transformer models, which process features independently, DynaGraph discovers feature connections through learnable parameters that generate time-specific adjacency matrices. This enables the model to capture evolving physiological patterns, for example, the sodium-potassium edge weight increases during electrolyte imbalance, while the creatinine-urea connection strengthens during acute kidney injury. Models were trained for up to 150 epochs using Adam optimisation with early stopping based on validation loss. All experiments used five random seeds for statistical robustness.

DynaGraph achieves state-of-the-art clinical prediction performance

We benchmark DynaGraph against two model families: (1) time-series architectures such as LSTM, GRU, TCN, Transformer, T-LSTM, RETAIN, BiT-MAC, and (2) graph-based methods such as GCN, GAT, TodyNet, SimTSC, and MedGNN. As shown in Table 1, DynaGraph consistently outperforms all baselines across AUROC, AUPRC, F1, and sensitivity.

On the MIMIC-III dataset, DynaGraph achieves an AUROC of 0.856, an AUPRC of 0.461, an F1 score of 47.04%, and a sensitivity of 85.22%, improving upon the strongest baseline (MedGNN) by +2.5 points in AUROC, +3.3 points in AUPRC, +0.96 points in F1, and a substantial +12.0% in sensitivity. On eICU, it reaches an AUROC of 0.802 and a sensitivity of 86.00%, with similarly strong gains in precision–recall. Comparable trends are observed on HiRID-ICU (AUROC 0.881, F1 59.37%, sensitivity 86.20%) and EHRSHOT (AUROC 0.849, F1 46.10%, sensitivity 79.44%), demonstrating robust generalisation across diverse clinical environments and temporal resolutions. While DynaGraph achieves significant relative improvements, the absolute

RETRACTED ARTICLE

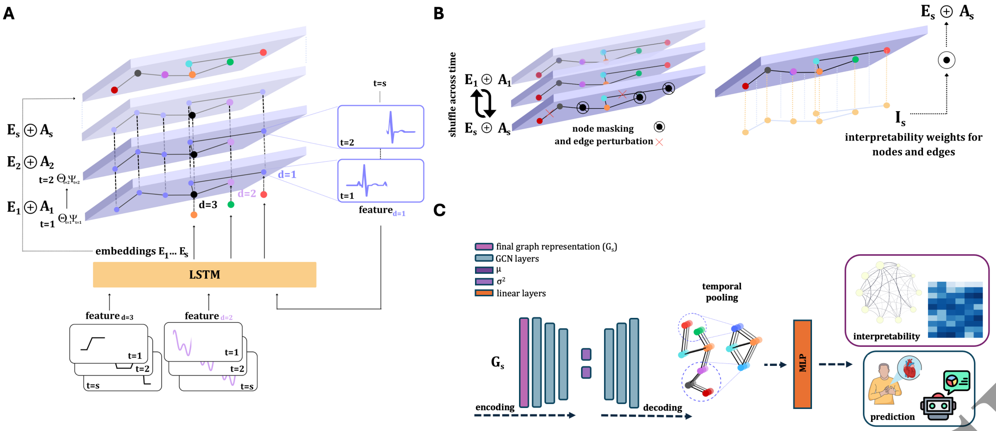

Fig. 1: Our DynaGraph model framework. The multivariate time-series $x_1, x_2, \ldots, x_d$ are divided into s equal-length time windows $t_1, t_2, \ldots, t_s$. **A:** Each window $t_1, t_2, \ldots, t_s$ has a corresponding feature matrix represented by a dynamic graph $A_1, A_2, \ldots, A_s$, whose adjacency matrices are learnable through information propagation. The node and edge vectors of the previous graph slice, θ_{t-1}, ψ_{t-1}, respectively, inform the corresponding nodes and edges of the next graph slice θ_t, ψ_t. The connections (expressed as dotted lines) between nodes within graph slices correspond to information propagation across time. An embedding matrix from the LSTM output $E_1, E_2, ..., E_s$ for the same time windows as for the graph construction ensures the pairing between the graphs and the temporal embeddings. **B:** The adjacency matrix is paired with an interpretability weight matrix I whose weights are learnable for every slice with a Hadamard product. The interpretability weights measure the impact of the different parts of the graph on the final loss $\mathcal{L}_{\text{total}}$. The adjacency matrix is also augmented with graph augmentation techniques for contrastive loss computation, such as shuffling across the s time dimension, random node masking, and edge perturbations. The adjacency matrix, interpretability matrix, and embeddings are aggregated to produce the final spatio-temporal graph representation of the multivariate time-series, namely $G_1, G_2, \ldots, G_s$. **C:** Finally, G_s is passed through a VGAE with GINs as the encoder-decoder and its output graph representation is clustered temporally with CNNs before being flattened for a standard MLP for multi-label classification.

F1 scores (45-59% across datasets) remain modest, reflecting fundamental challenges in clinical prediction. These values result from severe class imbalance in our multi-label setting, where positive outcomes range from 1.1% (peripheral vascular disease in eICU) to 38.1% (respiratory failure in HiRID). For rare outcomes like 30-day readmission (2.2% prevalence), even our best model achieves only 31% precision at 50% recall, highlighting the difficulty of identifying sparse positive cases amongst predominantly negative instances.

These improvements are driven by DynaGraph's joint modelling of temporal dynamics and relationships between clinical variables through dynamically evolving graphs. In contrast, baseline methods either flatten feature relationships (e.g., LSTM,

RETRACTED ARTICLE

Transformer) or rely on pre-structured or static graphs (e.g., GCN, MedGNN), limiting their ability to capture the time-sensitive structure of clinical events.

Table 1: Test results on different datasets for time-series and graph models in multi-label classification. AUROC and AUPRC are macro-averaged across all labels. Standard deviations are in parentheses. Higher is better. Best results are in bold. Results are averages of five random seeds.

Model	MIMIC-III				eICU			
	AUROC	AUPRC	F1	Sens	AUROC	AUPRC	F1	Sens
LSTM	0.721 (0.012)	0.287 (0.015)	38.46 (0.20)	47.12 (0.69)	0.682 (0.014)	0.198 (0.016)	30.07 (0.19)	39.58 (0.59)
GRU	0.748 (0.011)	0.324 (0.014)	47.55 (0.47)	42.70 (0.74)	0.664 (0.016)	0.187 (0.018)	29.91 (0.52)	30.09 (0.79)
Transformer	0.752 (0.010)	0.331 (0.013)	44.63 (0.46)	48.81 (0.79)	0.693 (0.013)	0.209 (0.015)	32.74 (0.48)	35.49 (0.78)
TCN	0.769 (0.009)	0.356 (0.012)	45.88 (0.47)	59.03 (0.88)	0.712 (0.012)	0.234 (0.014)	38.61 (0.40)	40.83 (0.77)
T-LSTM	0.743 (0.011)	0.318 (0.014)	40.50 (0.58)	48.92 (0.85)	0.708 (0.012)	0.227 (0.015)	32.48 (0.51)	40.76 (0.78)
RETAIN	0.768 (0.018)	0.354 (0.019)	45.97 (1.45)	66.30 (2.39)	0.707 (0.019)	0.229 (0.021)	36.00 (1.48)	40.10 (2.57)
BiT-MAC	0.749 (0.016)	0.329 (0.017)	43.60 (1.30)	47.25 (2.06)	0.697 (0.017)	0.221 (0.019)	35.55 (1.18)	39.44 (1.84)
PatchTST	0.783 (0.008)	0.371 (0.010)	45.91 (0.43)	63.57 (0.92)	0.728 (0.010)	0.248 (0.012)	39.23 (0.42)	45.92 (0.85)
GAT	0.734 (0.012)	0.306 (0.014)	41.79 (0.59)	47.16 (0.99)	0.719 (0.011)	0.218 (0.013)	31.84 (0.53)	36.74 (0.82)
GCN	0.754 (0.011)	0.334 (0.013)	42.83 (0.66)	51.24 (1.03)	0.741 (0.010)	0.236 (0.012)	32.96 (0.60)	37.91 (0.93)
TGAT	0.802 (0.007)	0.394 (0.009)	44.76 (0.38)	67.31 (0.87)	0.762 (0.008)	0.268 (0.010)	38.82 (0.39)	63.27 (0.89)
TodyNet	0.776 (0.008)	0.363 (0.011)	44.67 (0.25)	59.91 (0.73)	0.758 (0.007)	0.291 (0.009)	40.25 (0.26)	78.11 (0.75)
SimTSC	0.769 (0.013)	0.355 (0.015)	42.38 (0.42)	57.75 (1.15)	0.753 (0.012)	0.283 (0.013)	39.80 (0.44)	70.29 (1.31)
MedGNN	0.831 (0.006)	0.428 (0.008)	46.08 (0.28)	73.20 (0.94)	0.789 (0.006)	0.327 (0.008)	44.67 (0.37)	81.22 (0.99)
DynaGraph	**0.856 (0.005)*****	**0.461 (0.007)*****	**47.04 (0.23)**	**85.22 (0.76)**	**0.802 (0.005)****	**0.348 (0.007)*****	**45.96 (0.23)**	**86.00 (0.65)**

Model	HiRID-ICU				EHRSHOT			
	AUROC	AUPRC	F1	Sens	AUROC	AUPRC	F1	Sens
LSTM	0.827 (0.008)	0.418 (0.011)	42.48 (0.29)	67.10 (0.70)	0.673 (0.013)	0.256 (0.015)	31.91 (0.30)	53.40 (0.70)
GRU	0.821 (0.009)	0.426 (0.012)	44.11 (0.49)	66.60 (0.90)	0.679 (0.014)	0.263 (0.016)	32.24 (0.47)	54.73 (0.76)
Transformer	0.824 (0.009)	0.443 (0.013)	46.47 (0.63)	65.20 (0.80)	0.668 (0.015)	0.251 (0.017)	30.88 (0.49)	52.90 (0.80)
TCN	0.859 (0.007)	0.487 (0.010)	52.29 (0.57)	70.80 (0.80)	0.702 (0.011)	0.298 (0.013)	37.30 (0.45)	60.11 (0.85)
T-LSTM	0.831 (0.008)	0.423 (0.011)	43.27 (0.56)	69.00 (0.97)	0.723 (0.010)	0.324 (0.012)	40.08 (0.55)	64.82 (0.86)
RETAIN	0.847 (0.014)	0.479 (0.016)	51.89 (1.11)	69.27 (1.93)	0.754 (0.018)	0.356 (0.020)	42.33 (1.47)	67.96 (2.38)
BiT-MAC	0.835 (0.015)	0.461 (0.017)	49.31 (1.30)	67.10 (1.92)	0.748 (0.016)	0.348 (0.018)	41.23 (1.28)	66.14 (2.07)
PatchTST	0.864 (0.006)	0.498 (0.009)	53.86 (0.51)	73.42 (0.86)	0.778 (0.008)	0.371 (0.010)	43.17 (0.46)	70.28 (0.94)
GAT	0.794 (0.009)	0.387 (0.012)	40.95 (0.53)	61.30 (0.82)	0.715 (0.011)	0.309 (0.013)	37.82 (0.58)	62.39 (0.98)
GCN	0.799 (0.009)	0.395 (0.012)	41.80 (0.61)	66.50 (0.97)	0.718 (0.011)	0.321 (0.013)	39.74 (0.67)	63.21 (1.01)
TGAT	0.834 (0.007)	0.456 (0.010)	48.74 (0.45)	74.18 (0.91)	0.794 (0.007)	0.382 (0.009)	42.85 (0.40)	71.56 (0.93)
TodyNet	0.843 (0.007)	0.508 (0.009)	57.13 (0.40)	78.22 (0.89)	0.753 (0.007)	0.354 (0.009)	41.98 (0.26)	66.82 (0.76)
SimTSC	0.829 (0.012)	0.491 (0.013)	54.12 (0.51)	71.90 (1.20)	0.772 (0.012)	0.368 (0.013)	42.96 (0.41)	69.37 (1.14)
MedGNN	0.857 (0.005)	0.489 (0.008)	52.57 (0.41)	82.55 (1.07)	0.824 (0.005)	0.418 (0.007)	44.91 (0.27)	74.23 (0.95)
DynaGraph	**0.881 (0.004)*****	**0.527 (0.006)*****	**59.37 (0.27)**	**86.20 (0.83)**	**0.849 (0.004)*****	**0.447 (0.006)*****	**46.10 (0.24)**	**79.44 (0.71)**

**p < 0.01, ***p < 0.001 vs MedGNN (second best) using paired t-test across five random seeds.

Gradient-based attribution identifies time-specific feature importance patterns

A key strength of DynaGraph lies in its interpretability. The pseudo-attention mechanism provides slice-specific importance scores for graph components, enabling temporal attribution of predictive relevance. In Figure 2, we visualise attention heatmaps over time for the top ten features across datasets.

RETRACTED ARTICLE

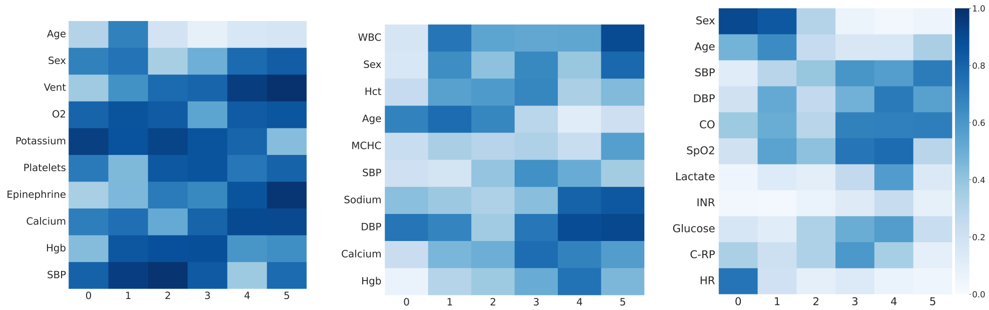

(a) MIMIC-III (b) eICU (c) HiRID-ICU

Fig. 2: Heatmaps of the pseudo-attention weight matrices for DynaGraph during training on (a) MIMIC-III, (b) eICU, and (c) HiRID datasets, highlighting the globally top 10 features. The x-axis represents the time-steps of ICU stay corresponding to 4-hour blocks and to temporal intervals of the constructed graphs. The weights are normalised and smoothed using a Gaussian kernel ($\sigma = 0.6$). Higher values indicate greater feature importance for the corresponding time-period in the final multi-label prediction tasks: heart attack complications (eICU), phenotype classification (MIMIC-III), and ICU mortality with heart/respiratory failure prediction (HiRID).

In MIMIC-III and eICU, early windows place relatively greater weight on static covariates (age, sex) for initial risk stratification, with laboratory variables (e.g., sodium, WBC, haemoglobin) generally increasing in prominence from mid to later windows. Patterns are dataset-specific; however, for example, sex remains among the top contributors in later eICU windows, and potassium exhibits a mid-course peak rather than a monotonic increase. These variable behaviours have well-established relevance in ICU deterioration and cardiovascular risk [28–30].

Graph-level individual patient snapshots (Figure 3) illustrate how the model reconfigures its attention across time: node sizes reflect feature contributions to the loss, while edge weights capture temporal dependencies. These evolving topologies offer intuitive explanations aligned with clinical progression.

The structural changes in Figure 3 reveal clinically interpretable dynamics. At $t = 1$ (admission), the Vent (mechanical ventilation) node exhibits strong connections to multiple physiological variables, reflecting the central role of respiratory support in early ICU management. By $t = 6$, these connections weaken substantially, a pattern consistent with successful weaning from mechanical ventilation, a common trajectory for improving patients. Concurrently, edges between renal markers (creatinine, calcium) and electrolytes (potassium) strengthen during mid-stay windows, reflecting the development of cardiorenal interactions frequently observed in critical illness [31]. The emergence of haemoglobin (Hgb) and systolic blood pressure (SBP) as prominent nodes at $t = 4$–6 aligns with the clinical importance of haemodynamic stability and oxygen delivery in determining ICU outcomes. While these patterns demonstrate that DynaGraph learns physiologically plausible temporal dynamics, formal causal validation remains an important direction for future work. The temporal evolution of feature importance aligns with establishe

RETRACTED ARTICLE

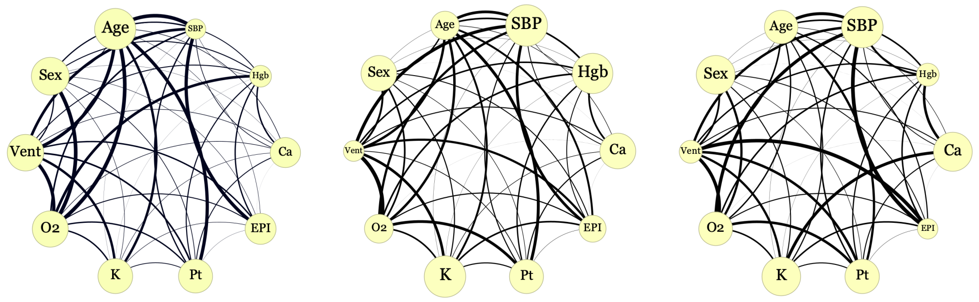

(a) t=1 (b) t=4 (c) t=6

Fig. 3: Learned graph representations during DynaGraph training on the MIMIC-III dataset at three distinct timesteps: (a) $t = 1$, (b) $t = 4$, and (c) $t = 6$, each corresponding to the first, fourth, and last 4-hour interval of a 24-hour ICU stay. The visualisation highlights the evolving patterns captured by the model, including changes in individual feature importance and correlations between features. Node size corresponds to node weight magnitude, while edge darkness reflects edge weight magnitude, as derived from the interpretability matrices. These representations demonstrate the model's ability to dynamically adapt to temporal changes in the data.

Ablation studies highlight importance of each model component

To understand the contribution of each module, we ablate six components: (1) graph augmentations and contrastive loss (AUG), (2) focal loss (FOC), (3) LSTM embeddings, (4) regularisation (REG), (5) structural loss (STRUC), and (6) temporal graph pooling (TGP). Results (Figure 4) demonstrate that removing any single module degrades AUROC performance, with the most substantial drops from contrastive augmentation (-0.048 AUROC) and focal loss (-0.035 AUROC).

The ablation results support our design choices, which show that removing contrastive augmentation (AUG) causes the largest performance degradation across all datasets (AUROC drops of 0.048, 0.046, 0.042, and 0.044 for eICU, MIMIC-III, HiRID-ICU, and EHRSHOT, respectively), confirming its necessity for learning robust representations from sparse, irregularly-sampled clinical data. Focal loss removal (FOC) results in the second-largest impact with AUROC reductions of 0.035-0.038 across datasets, demonstrating its critical role in handling severe class imbalance where positive outcomes range from 2-38% prevalence. LSTM embeddings contribute 0.025-0.030 AUROC, validating the importance of capturing within-feature temporal patterns alongside graph-based inter-feature relationships. Regularisation (REG) and temporal graph pooling (TGP) show moderate but consistent contributions (0.020-0.025 AUROC), whilst structural loss (STRUC), though showing the smallest individual impact (0.014-0.018 AUROC), prevents training instability by ensuring smooth transitions in adjacency matrices between consecutive time windows.

RETRACTED ARTICLE

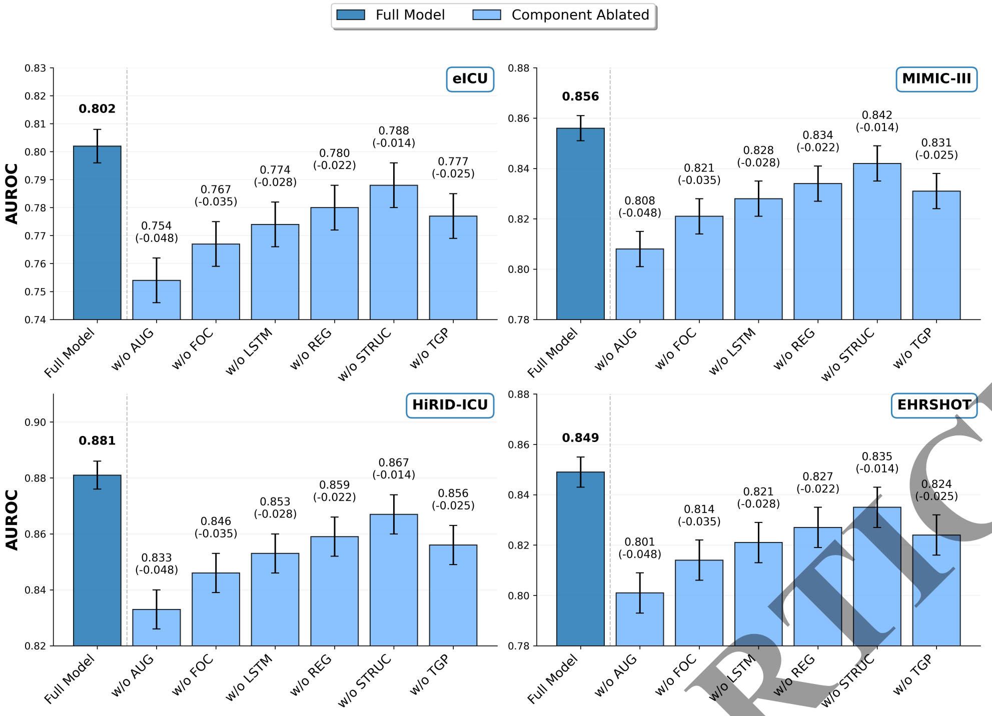

Fig. 4: Ablation study showing the impact of removing individual components on AUROC performance across four datasets: (a) eICU, (b) MIMIC-III, (c) HiRID-ICU, and (d) EHRSHOT. The Full Model bar (dark blue) represents the complete DynaGraph architecture with all components, while the subsequent bars (light blue) show performance when each component is removed. Components ablated include: AUG (graph augmentation and contrastive loss), FOC (focal loss), LSTM (LSTM embedding module), REG (regularisation loss), STRUC (structural loss), and TGP (temporal graph pooling). Values above each bar indicate the AUROC score, with drops from the full model shown in parentheses for ablated components. Error bars represent standard deviation across five random seeds. The vertical dashed line separates the reference full model from the ablation results.

Robust generalisation in out-of-distribution (OOD) settings

We evaluate DynaGraph's generalisability by constructing out-of-distribution (OOD) test sets: age groups (< 20, > 80), sex, and ICU time quartiles (Q1 to Q4). As shown in Figure 5, DynaGraph maintains high performance across all subgroups, outperforming MedGNN in every OOD scenario.

Performance remains strong even in challenging strata, such as young patients and early ICU windows, thus underscoring DynaGraph's ability to generalise beyond its training distribution. This robustness is critical for real-world deployment in heterogeneous healthcare populations.

RETRACTED ARTICLE

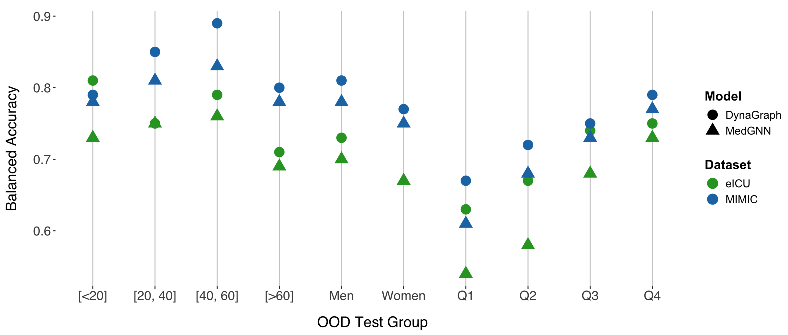

Fig. 5: Generalisation performance of DynaGraph and MedGNN on out-of-distribution (OOD) settings, evaluated using balanced accuracy. OOD test sets were constructed by excluding specific subgroups like age groups: < 20 to > 80; gender: male and female; and time quartiles: Q1 (first 6 hours of stay) to Q4 (last 6 hours of stay) from the training data on the eICU and MIMIC-III datasets. DynaGraph demonstrates superior robustness across all OOD scenarios compared to MedGNN. Note: The result for MedGNN for "Women" under the eICU setting is behind the indicator for DynaGraph for "Women" under the MIMIC setting, as they both equal 0.77 balanced accuracy.

Discussion

Accurately modelling clinical time-series data is one of the central challenges in machine learning for healthcare. While recent deep learning methods such as recurrent networks, Transformers, and static graph models have improved predictive performance on EHR datasets, most treat features as independent and assume fixed structures or uniform time dependencies. These assumptions obscure the dynamic and relational nature of physiological processes, limiting both model accuracy and clinical interpretability.

DynaGraph advances beyond recent temporal modelling approaches in healthcare. While CLOCS [32] achieves strong performance through contrastive learning on static snapshots, it cannot capture evolving feature relationships. STraTS [33] handles irregular sampling through neural ODEs but processes features independently, missing relationships between different clinical variables (cross-variable dependencies). Our dynamic graph learning addresses the limitation identified by [34] that "static architectures fail to model the non-stationary dynamics of physiological systems." Work by Rocheteau et al. [35] on temporal pointwise convolutions and Horn et al. [36] on set functions for irregular time-series still assumes fixed feature relationships throughout patient trajectories.

DynaGraph learns spatio-temporal graphs end-to-end from raw multivariate EHR time-series without making any structural assumptions, capturing how relationships between clinical features evolve over time. DynaGraph tracks two types of patterns: (1) how individual clinical variables change over time within each patient (e.g., creatinine rising from 1.2 to 3.5 mg/dL over 24 hours), and (2) how relationships between

RETRACTED ARTICLE

different variables evolve (e.g., the correlation between blood pressure and urine output strengthening during shock). The model achieves this by combining sequential processing (LSTM) for within-variable trends with graph learning for between-variable relationships. The interpretability mechanism uses gradient magnitudes to quantify which variable pairs contribute most to predictions at each interval. This enables both strong predictive performance and clinically meaningful explanations.

Our results demonstrate that DynaGraph consistently outperforms state-of-the-art time-series and graph-based models across diverse tasks and care settings. Notably, it delivers substantial gains in AUPRC (6-8% relative improvement) in highly imbalanced multi-label settings, a common reality in healthcare. The inclusion of contrastive graph augmentation, focal loss, and structural regularisation further enhances robustness, especially in out-of-distribution scenarios involving age, sex, and ICU stage variability.

DynaGraph surfaces time-resolved insights into the evolving relevance of clinical features. The learned pseudo-attention weights align with established risk markers such as sodium, potassium, and haemoglobin while adapting across stages of a patient's trajectory. The temporal evolution of feature importance aligns with established clinical pathophysiology while uncovering novel patterns. The early emphasis on static demographics followed by transition to dynamic markers reflects clinical risk stratification practices described in the Surviving Sepsis Campaign guidelines [37]. The intensification of sodium-potassium edge weights at hours 12-16 corresponds to the typical onset of dysnatraemia in critical illness, occurring in 30-40% of ICU patients [38]. Notably, we observed the creatinine-haemoglobin coupling strengthened 8-10 hours before conventional AKI criteria, suggesting potential for earlier intervention aligned with findings that subclinical kidney stress precedes creatinine elevation by 12-24 hours [39]. Visualisations of individual patient graphs reveal dynamic patterns of physiological importance, and computing feature-pair importance scores via gradient magnitudes (Equations 3-5) can quantify each edge's contribution to loss at 3-hour intervals

DynaGraph addresses three critical gaps in current clinical decision support identified by Topol [40]. First, it provides temporal granularity, clinicians can see not just that a patient is deteriorating, but which organ system interactions are driving risk. Second, the interpretability mechanism enables "diagnostic reasoning transparency" [41], showing why specific timepoints matter. Third, by learning patient-specific graphs, it supports precision medicine approaches where treatment targets the dominant pathophysiological mechanism [42]. DynaGraph's 13ms inference time enables integration into existing clinical decision support systems at multiple touchpoints: automated early warning scores during nursing rounds, dynamic risk stratification during physician handoffs, and continuous monitoring in intensive care settings. The interpretability mechanism provides actionable insights by highlighting which physiological systems require immediate attention. For example, when the model elevates renal markers (creatinine, BUN) while maintaining low cardiac weights, it may suggest isolated kidney injury rather than cardiorenal syndrome, guiding targeted intervention.

Real-world deployment faces several challenges [43]. Computational requirements (13ms inference on GPU) necessitate local edge computing or cloud infrastructure with sub-second latency. Data harmonisation across institutions remains problematic, while we used common features (vital signs, basic labs), site-specific normal ranges

RETRACTED ARTICLE

require calibration [44]. The distinction between our "framework" and a "model" is deliberate: DynaGraph provides an architectural paradigm for learning dynamic clinical graphs that can be instantiated with different backbone networks (GIN, GAT, GraphSAGE), similar to how ResNet defined a framework instantiated across vision tasks [45]. Hospitals could adapt the framework to local feature sets while preserving the core dynamic learning mechanism.

Compared to recent multimodal or pretrained frameworks (e.g., COMET [46]), which rely on external omics or large-scale transfer learning, DynaGraph uses solely EHR data and learns structure directly from temporal dynamics. This enables broader applicability to settings where curated labels or external modalities are unavailable. Moreover, unlike foundation models that treat the EHR as flat token sequences, DynaGraph embeds inductive biases aligned with clinical reasoning, thereby emphasising evolving relationships between physiological signals.

Despite its strengths, DynaGraph has several limitations. First, its interpretability mechanism, though grounded in gradients and attention-like weighting, does not provide causal guarantees. Distinguishing "sodium causes potassium changes" from "both reflect kidney dysfunction" requires causal inference methods [47]. Future work may incorporate counterfactual analysis or causal inference techniques to enhance explanatory depth. Second, while we mitigate computational overhead through temporal pooling, dynamic graph construction and contrastive training remain resource-intensive. Exploring pruning, distillation, or sparsity-aware architectures could improve scalability. Third, the 24-hour window may miss longer-term patterns relevant for chronic disease management [48]. Fourth, Black patients and other minorities were underrepresented (8% in MIMIC-III), raising fairness concerns identified by Obermeyer et al. [49]. Recent implementation studies show model performance degrades 5-8% when deployed prospectively due to temporal dataset shift [50], our contrastive augmentation partially addresses this through improved generalisation. Finally, we have not yet explored deployment in streaming or real-time clinical systems, an important direction for future translational work.

Looking ahead, we envision three key extensions of DynaGraph. First, incorporating additional data modalities such as free-text notes, radiographic features, or genomics into a unified dynamic graph could enable richer clinical representations. Second, human-in-the-loop workflows could allow clinicians to adjust, annotate, or audit learned graph structures, fostering trust and feedback-driven improvement. Third, privacy concerns could be addressed through federated learning extensions, training local graph structures without sharing patient data [51].

In conclusion, DynaGraph advances a new direction in clinical machine learning: dynamic, interpretable graph learning tailored to the structure of EHR data. By modelling evolving feature relationships and delivering time-resolved insights, it achieves AUROC 0.856-0.881 while identifying which of the numerous possible feature pairs contribute most to predictions at each time window. We believe this work brings us closer to the deployment of transparent, trustworthy, and generalisable AI systems in healthcare.

RETRACTED ARTICLE

Methods

Data

We evaluated our proposed framework on four publicly available longitudinal electronic health record (EHR) datasets that include both ICU and primary care settings. The MIMIC-III v1.4 ICU dataset comprises 53,423 distinct hospital admissions [52]. Following the preprocessing pipeline proposed by [53], we extract a subcohort of 17,279 ICU admissions (from 11,563 unique patients), each characterised by static variables (age and sex) and 42 laboratory measurements in time-series. The prediction tasks include ten binary classification outcomes: ICU mortality (7.65% positive cases), hospital mortality (9.65%), 30-day readmission (2.19%), shock (7.57%), acute cerebrovascular disease (8.99%), acute myocardial infarction (10.26%), cardiac dysrhythmias (32.60%), chronic kidney disease (12.14%), chronic obstructive pulmonary disease and bronchiectasis (11.82%), and congestive heart failure (24.15%). The eICU Collaborative Research Database is a multi-centre ICU dataset containing over 200,859 patient unit encounters from 139,367 unique patients across 335 ICUs in 208 hospitals in the United States [44]. We focus on a subset of heart attack patients admitted to critical care units, such as the coronary care unit (CCU), who were monitored for potential complications. Among these patients, 16.00% developed at least one of the following complications: peripheral vascular disease (1.10%), heart failure (10.26%), atrial fibrillation (28.49%), arrhythmias (23.14%), or death (12.00%) [54]. The HiRID-ICU dataset includes more than 33,000 patient admissions to an intensive care unit. We used the imputed staging dataset provided by the original investigators, with prediction tasks that focused on ICU mortality (8.39%), respiratory failure (38.12%), and circulatory failure (3.10%) [55, 56]. EHRSHOT is based on primary care data from the Stanford Medicine Research Data Repository and Stanford Health Care of 6,739 patients [57]. After removing patients with fewer than 24 time-stamped measurements, no time-series vital signs or laboratory values, outliers, and no missing labels of interest, 2,378 remain. Our labels include the first occurrence of heart attack (9.52%), lupus (5.73%), celiac disease (3.45%), pancreatic cancer (9.30%), hyperlipidemia (14.84%), and hypertension (16.39%) after 1 year post-discharge.

Outliers were detected using a 5-standard deviation threshold from the mean and replaced with the 5σ boundary value to prevent extreme values from dominating gradient updates while preserving clinically relevant variations (full preprocessing pipeline in Supplementary Note 1). For irregularly sampled time-series, we applied forward-filling for up to 6 hours, followed by patient-specific median imputation, then global median for remaining missingness. Features with ¿70% missing values were excluded. Time-series were resampled to hourly intervals (5-minute for HiRID-ICU) using forward-fill to align with the clinical practice of carrying forward the last known values.

For MIMIC-III, patients with multiple ICU stays had each admission treated as independent if separated by ¿48 hours, following standard preprocessing protocols. Patient-level splitting ensured no patient appeared in multiple sets: the 80:10:10 train-validation-test split was stratified by primary outcome with explicit verification that patient IDs were unique across splits, preventing data leakage (Supplementary Note 2).

RETRACTED ARTICLE

Hyperparameter optimisation used grid search on the validation set only, as detailed in Supplementary Note 2. The optimal configuration (batch=128, lr=0.001, windows=6, dropout=0.5) was fixed for all test set evaluations.

Dynamic Graph Construction

Figure 1 shows the overall framework of DynaGraph. Input is defined as a collection of multivariate time-series measurements for N patients. A patients is then described by $X = \{x_1, x_2, \ldots, x_d\} \in \mathbb{R}^{d\times l}$ with d features of length l. Given a group of m patients $X = \{X_1, X_2, \ldots, X_m\} \in \mathbb{R}^{m\times d\times l}$, their corresponding labels $Y = \{y_1, y_2, \ldots, y_m\}$ mean y is a predefined class vector of binary labels for each patient, and $m \in \mathbb{N}^*$. The final graph construction consists of three components:

Graph Construction Through Information Propagation.

We dissect the time-series into s equal-sized time-windows $T = [t_1, t_2, \ldots, t_s]$, X now becomes $X \in \mathbb{R}^{d\times l\times s}$, where the first window t_1 is used to construct an initial static graph representation with nodes representing the time-series features and the edges the hidden associations between the features. The graph representation is captured in an adjacency matrix with the rows and columns corresponding to the features and nodes, respectively. All elements of the adjacency matrix are learnable parameters in the model, initialised randomly. Each node is assigned two values, the source and target nodes. We generate vectors Θ and Ψ with length d (number of features) for each time window t, and all elements are learnable parameters that are initialised randomly. The initial adjacency matrix is then the multiplication of these vectors for a time slot:

$$A = \Theta^{\mathrm{T}} \cdot \Psi \in \mathbb{R}^{d\times d} \tag{1}$$

where $\Theta = [\theta_{t,1}, \theta_{t,2}, \ldots, \theta_{t,d}]$, $\Psi = [\psi_{t,1}, \psi_{t,2}, \ldots, \psi_{t,d}]$ represent the random initialization of learnable node embeddings. The adjacency matrix is made more sparse to reduce the computational costs by using the top-k largest values of the adjacency matrix:

$$\begin{gathered} idx, idy = \mathrm{argtopk}(A[:,,]) \quad idx \neq idy \\ A[-idx, -idy] = 0 \end{gathered} \tag{2}$$

Subsequent time windows are used to construct dynamic graph representations, aggregated node-wise through message passing. For each time slot, new vertices are added to represent features from the previous time slot, resulting in a set of vertices $\{v_{(t,1)}, v_{(t,2)}, \ldots, v_{(t,D)}, v_{(t-1,1)}, v_{(t-1,2)}, \ldots, v_{(t-1,D)}\}$. The edges are directed from previous time vertices to their counterparts in the current time window, connecting $v_{(t-1,d)}$ to $v_{(t,d)}$ for $d = 1, 2, \ldots, D$ where D is the total number of features or nodes. Since the number of features, nodes, or vertices does not change over time, the new set of nodes is connected to the previous set, signifying the addition of time connections in the graph. To prevent an exponential increase in the number of nodes, new node embeddings are aggregated, and redundant vertices are removed. The graph representation is a set of adjacency matrices $A = \{A_1, A_2, \ldots, A_s\} \in \mathbb{R}^{d\times d\times s}$ with d features or nodes and for s time-windows capturing spatio-temporal patterns in the multivariate time-series.

RETRACTED ARTICLE

While this formulation is data-driven with random initialisation, the adjacency matrix learns clinically meaningful relationships through several mechanisms. First, the parameters Θ and Ψ are optimised end-to-end via backpropagation to minimise clinical prediction objectives (mortality, complications), ensuring that discovered edges represent physiologically relevant interactions predictive of patient outcomes. Second, our multi-component loss function guides the learning process. Third, as validated later, the learned graphs can consistently identify established clinical relationships while potentially discovering novel predictive patterns. This data-driven approach offers advantages over fixed clinical graphs by enabling temporal adaptation of relationships and personalisation to patient trajectories.

LSTM Embeddings.

$X \in \mathbb{R}^{d\times l\times s}$ is also, in parallel, processed by a Long-Short Term Memory (LSTM) unit to derive an embedding matrix for each time slice, encapsulated as $E = \{E_1, E_2, ..., E_s\} \in \mathbb{R}^{d\times d\times s}$. The LSTM embeddings help capture the long-term within-variable temporal patterns of individual time-series, whereas the graph learns efficient relationships between different clinical variables in discrete time slices.

Interpretability.

We used a paired weight matrix for each graph with learnable weights that would update itself based on the contribution of each node or edge of the adjacency matrix to the total loss. The adjacency matrix of each graph is paired with a uniformly initialised weight matrix I of the same dimension $d \times d \times s$ whose weights are updated depending on which node or edge contributes the most to the downstream loss computation. This allows us to use the weights in this matrix as 'pseudo-attention' weights, telling us which parts of the adjacency matrix, and thus the graph, contributed the most to the prediction.

We define a set of interpretability weight matrices $I = \{I_1, I_2, \ldots, I_s\} \in \mathbb{R}^{d\times d\times s}$ on top of the adjacency matrices for each time slice whose weights are updated based on the graph nodes or edges contributing to the loss gradient update.

The total contribution of a feature (node) v, I_v, is quantified by combining its direct importance with the average importance of its connections (edges) to other nodes. This can be expressed as:

$$I_v = \alpha \cdot I_{vv} + (1 - \alpha) \cdot \bar{I}_{vu} \tag{3}$$

where α is a balancing parameter. The node and edge importances are elements in the interpretability matrix and are computed as follows:

$$I_{vv} = \|\nabla_{h_v} L\| \tag{4}$$

$$I_{vu} = \|\nabla_{e_{v,u}} L\| \tag{5}$$

where h_v denotes the feature vector of node v, $e_{v,u}$ represents the edge between nodes v and u, and L is the loss function of the model. Average Edge Importance$_v$ is the average of the gradients for the edges connected to node v. After iteration, the final values

RETRACTED ARTICLE

of the importance weights are normalised across all features to ensure comparability. We provide a proof of how the interpretability weight matrix I converges under certain loss and learning rate assumptions in Supplementary Note 4. Graph Isomorphism Networks (GINs) [58] represent a significant advancement in graph neural networks by approximating the Weisfeiler-Lehman (WL) test through its learnable framework. GINs effectively learn powerful node embeddings that capture the topology of the graph, making it possible to distinguish between different graph structures.

The update rule for GIN is given by:

$$h_v^{(k)} = \mathrm{MLP}^{(k)} \left((1 + \epsilon^{(k)}) h_v^{(k-1)} + \sum_{u \in \mathcal{N}(v)} h_u^{(k-1)} \right) \tag{6}$$

where $h_v^{(k)}$ is the node representation of v at layer k, $\mathcal{N}(v)$ denotes the neighbours of v, $\epsilon^{(k)}$ is a learnable parameter that adjusts the weighting of the node's own features relative to its neighbours, and $\mathrm{MLP}^{(k)}$ is a multi-layer perceptron. In DynaGraph, the GIN is used to obtain graph representations at the latent level for mean and variance computation in variational inference for the VGAE as well as a decoder for the final graph output before temporal pooling for downstream tasks.

In the context of DynaGraph, we propose a novel approach to enhance the interpretability of predictions in EHR multivariate time-series data. Our model employs a pseudo-attention mechanism that operates on the graph construction phase. This mechanism allows the model to dynamically adjust the graph's structure, thus enhancing the interpretability of the learned representations. The interpretability function for a node v, for example, can be expressed mathematically as:

$$\mathrm{Score}_v = \sum_{k=1}^{K} \|\mathrm{grad}(y, h_v^{(k)})\|_2 \tag{7}$$

where y is the prediction output, and $\mathrm{grad}(y, h_v^{(k)})$ represents the gradient of the loss function at y with respect to the node embedding $h_v^{(k)}$. This gradient highlights the influence of each node on the prediction, allowing clinicians and researchers to identify critical features in the graph that significantly impact patient outcomes.

Moreover, by integrating this interpretability function directly into the dynamic graph learning process, DynaGraph not only adapts to changes in data over time but also provides insights into how these changes influence predictive outcomes. This method bridges the gap between complex model predictions and clinical decision-making by providing a transparent view of feature importance over time.

The adjacency, embedding, and interpretability matrices are aggregated for a final graph representation G:

$$G^{(i)} = (A^{(i)} + E^{(i)}) \odot I^{(i)} \tag{8}$$

where $+$ denotes element-wise addition and $\odot$ denotes the Hadamard (element-wise) product. We define interpretability as the ability to quantify each feature pair's contribution to the final prediction through gradient-based importance scores

RETRACTED ARTICLE

$I_{vu} = \|\nabla_{e_{v,u}} L\|$, where higher magnitudes indicate stronger influence on the loss function. The embeddings are first added to the adjacency matrices to combine structural and temporal information, then the resulting matrix is element-wise multiplied by the interpretability weights to modulate edge importance. This final representation captures both the temporal patterns within individual time-series from the LSTM and the spatio-temporal patterns between time-series features from the graph construction, weighted by their learned importance for the prediction task.

The interpretability mechanism serves dual purposes: providing transparency for clinical decision-making and validating that learned relationships align with physiological understanding. By examining which edges and nodes contribute most to predictions (through gradient magnitudes in Equations 4-5), clinicians can verify that the model prioritises clinically sensible relationships. This interpretability framework acts as a safeguard against purely spurious correlations that might emerge from unconstrained data-driven learning, ensuring that DynaGraph's predictions are both accurate and clinically grounded. Implementation details are described further in Supplementary Note 3.

GIN encoder and VGAE.

Let $H_t^{(0)} \in \mathbb{R}^{d \times p}$ denote the per–time-slice node features from the LSTM pathway for slice t. To align with the $(A+E) \odot I$ fusion defined above, we instantiate the slice-level embedding affinity

$$E_t^{(i)} := \mathrm{norm}\big(H_t^{(0)} H_t^{(0)^\top}\big) \in \mathbb{R}^{d \times d},$$

and form the final slice graph

$$G_t^{(i)} \;=\; \big(A_t^{(i)} + E_t^{(i)}\big) \,\odot\, I_t^{(i)} \;\in\; \mathbb{R}^{d \times d}.$$

We then obtain slice-wise node representations with a GIN. The GIN update is

$$h_{v,t}^{(k)} \;=\; \mathrm{MLP}^{(k)} \left((1 + \epsilon^{(k)})\, h_{v,t}^{(k-1)} + \sum_{u \in \mathcal{N}_{G_t}(v)} h_{u,t}^{(k-1)} \right), \tag{9}$$

where $\mathcal{N}_{G_t}(v)$ are neighbors of v in $G_t^{(i)}$, $\epsilon^{(k)}$ is learnable, and $h_{v,t}^{(0)}$ is the vth row of $H_t^{(0)}$. Stacking node embeddings yields $H_t^{(K)} \in \mathbb{R}^{d \times q}$.

For variational inference, a GIN-based encoder maps $(G_t^{(i)}, H_t^{(0)})$ to Gaussian parameters:

$$\mu_t^{(i)},\ \log \sigma_t^{2\,(i)} \;=\; \mathrm{GIN}_{\mathrm{enc}}\big(G_t^{(i)}, H_t^{(0)}\big), \tag{10}$$

and we sample latent node codes via the reparameterization trick,

$$Z_t^{(i)} \;=\; \mu_t^{(i)} + \sigma_t^{(i)} \odot \epsilon, \qquad \epsilon \sim \mathcal{N}(0, I), \tag{11}$$

with $Z_t^{(i)} \in \mathbb{R}^{d \times q}$. A GIN-based decoder reconstructs the slice graph:

$$\widehat{G}_t^{(i)} \;=\; \sigma\Big(\mathrm{GIN}_{\mathrm{dec}}\big(Z_t^{(i)}\big)\Big), \tag{12}$$

RETRACTED ARTICLE

where $\sigma(\cdot)$ is the element-wise sigmoid. The VGAE loss sums across slices:

$$\mathcal{L}_{\mathrm{VGAE}} \;=\; \sum_{t=1}^{s} \Big[\; \mathrm{BCE}\big(G_t^{(i)},\, \widehat{G}_t^{(i)}\big) \;+\; \mathrm{KL}\big(\mathcal{N}(\mu_t^{(i)},\, \sigma_t^{2\,(i)}) \,\|\, \mathcal{N}(0,I)\big) \;\Big]. \tag{13}$$

Temporal pooling.

To avoid flattening and preserve structure, we apply hierarchical, differentiable pooling over nodes (shared across time slices). Let $N^{(\ell)}$ be the number of nodes at pooling layer ℓ. A 2D convolutional module produces cluster activations and an assignment matrix $M^{(\ell)} \in \mathbb{R}^{N^{(\ell+1)} \times N^{(\ell)}}$. Given slice-$t$ graph $G_t^{(\ell)}$ and node embeddings $X_t^{(\ell)}$, we compute

$$X_t^{(\ell+1)} = \mathrm{CNN}^{(\ell)}\big(X_t^{(\ell)}\big), \tag{14}$$

$$G_t^{(\ell+1)} = M^{(\ell)} G_t^{(\ell)} M^{(\ell)^{\top}}, \tag{15}$$

with $G_t^{(\ell+1)} \in \mathbb{R}^{N^{(\ell+1)} \times N^{(\ell+1)}}$. Here $M^{(\ell)}$ is a learned soft assignment (rows sum to 1), yielding coarser graphs that retain edge patterns. After L_p pooling stages we obtain $\{G_t^{(L_p)}, X_t^{(L_p)}\}_{t=1}^{s}$, which are vectorised and passed to the downstream MLP classifier for multi-label prediction.

Graph Augmentation, Model Training and Interpretation

We introduce graph augmentations to help our model generalise and learn temporal variability. We couple this with a contrastive loss, which encourages the model to capture both spatial (inter-node) and temporal (intra-node) dependencies. Augmentations include shuffling along the time axis and perturbing the adjacency matrix A through node dropping or edge perturbations, creating an augmented graph pair A_+. A negative sample A_- is also created for each original graph pair A. The augmented and negative samples are compared to the original graph using cosine similarity, forming the contrastive loss:

$$\mathcal{L}_{\mathrm{contrast}} = -\mathbb{E}\left[\log \frac{e^{\mathrm{Sim}_\theta(A,A_+)}}{e^{\mathrm{Sim}_\theta(A,A_+)} + \sum_{A_-} e^{\mathrm{Sim}_\theta(A,A_-)}}\right] \tag{16}$$

Sim_θ represents the cosine similarity $\mathrm{Sim}_\theta\left(\boldsymbol{x}_{n,i}, \boldsymbol{x}_{n,j}\right) = \boldsymbol{x}_{n,i}^{\top} \boldsymbol{x}_{n,j} / \left\|\boldsymbol{x}_{n,i}\right\| \left\|\boldsymbol{x}_{n,j}\right\|$ for the pair of nodes i, j of the nth graph or adjacency matrix in the minibatch.

To address the class imbalance in multi-label prediction, we employ a focal loss:

$$\mathcal{L}_{\mathrm{focal}}\left(\widehat{y}\right) = -(1-\widehat{y})^{\gamma} \cdot \log(\widehat{y}), \quad \gamma \geq 0 \tag{17}$$

where the γ parameter accounting for the class weighting is considered as a hyperparameter.

RETRACTED ARTICLE

A regularisation term penalises large variations in node features to preserve smoothness:

$$\mathcal{L}_{\text{reg}} = \lambda \sum_{(i,j)\in E} \|\mathbf{h}_i - \mathbf{h}_j\|^2 \tag{18}$$

where λ is a hyperparameter controlling the strength of the regularization, (i, j) represents an edge connecting nodes i and j, and $\mathbf{h}_i$, $\mathbf{h}_j$ are the feature representations of nodes i and j, respectively.

Additionally, we define a structural similarity loss to ensure that the learned graph structure remains close to the original (previous timepoint) structure:

$$\mathcal{L}_{\text{structure}} = \mu \left(1 - \frac{\sum_{i,j} A_{ij} \cdot A'_{ij}}{\sqrt{\sum_{i,j} A_{ij}^2} \cdot \sqrt{\sum_{i,j} A'^2_{ij}}}\right) \tag{19}$$

where A is the original adjacency matrix, A' is the adjacency matrix after augmentation, and μ is a hyperparameter. The structural loss aids in the convergence of the adjacency matrix throughout learning. The final loss is the sum of these losses where the balancing parameters ϵ, λ, μ, and β are considered as hyperparameters:

$$\begin{aligned}\mathcal{L}_{\text{total}} = \alpha\mathcal{L}_{\text{contrast}} + \epsilon\mathcal{L}_{\text{focal}} + \lambda\mathcal{L}_{\text{reg}} + \\ \mu\mathcal{L}_{\text{structure}} + \beta\mathcal{L}_{\text{VGAE}}\end{aligned} \tag{20}$$

As described earlier in this section, focal loss is a common adjustment made in cases of strong class imbalance as in our healthcare scenarios. Contrastive loss aids in predictive performance by allowing for more diverse or augmented representations of the input graphs in learning downstream tasks. The regularisation loss term is added to reduce overfitting and stabilise the loss within bounds. The structural loss ensures that next time-step graph representations do not deviate too far from the previous representation to stabilise the dynamic graphs through time.

After integrating the embeddings with the augmentations, G_s is passed through a variational graph autoencoder (VGAE) whose encoder-decoder structure follows a GIN architecture. The latent features are graph representations of the mean and variance vectors. The output of the VGAE is a graph reconstruction, which is pooled temporally to reduce the number of nodes with dynamic clustering and decrease the computational costs of the training. The reduced graph is then flattened and passed through a multilayer perceptron for the final multilabel classification.

Data Availability. Data is available via application to https://physionet.org/content/mimiciii/1.4/, https://eicu-crd.mit.edu/gettingstarted/access/, https://physionet.org/content/hirid/1.1.1/, and https://redivis.com/datasets/53gc-8rhx41kgt.

Code availability. Code for running and analysing the models can be found here: https://github.com/munibmesinovic/DynaGraph.git.

RETRACTED ARTICLE

References

[1] Siontis, K.C., Noseworthy, P.A., Attia, Z.I., Friedman, P.A.: Artificial intelligence-enhanced electrocardiography in cardiovascular disease management. Nature Reviews Cardiology **18**(7), 465–478 (2021)

[2] Smith, M.B., Chiovaro, J.C., O'Neil, M., Kansagara, D., Quiñones, A.R., Freeman, M., Motu'apuaka, M.L., Slatore, C.G.: Early warning system scores for clinical deterioration in hospitalized patients: a systematic review. Annals of the American Thoracic Society **11**(9), 1454–1465 (2014)

[3] Shamout, F., Zhu, T., Clifton, D.A.: Machine learning for clinical outcome prediction. IEEE reviews in Biomedical Engineering **14**, 116–126 (2020)

[4] Lauritsen, S.M., Kristensen, M., Olsen, M.V., Larsen, M.S., Lauritsen, K.M., Jørgensen, M.J., Lange, J., Thiesson, B.: Explainable artificial intelligence model to predict acute critical illness from electronic health records. Nature communications **11**(1), 3852 (2020)

[5] Harutyunyan, H., Khachatrian, H., Kale, D.C., Ver Steeg, G., Galstyan, A.: Multitask learning and benchmarking with clinical time series data. Scientific data **6**(1), 96 (2019)

[6] Sheikhalishahi, S., Balaraman, V., Osmani, V.: Benchmarking machine learning models on eicu critical care dataset. arXiv preprint arXiv:1910.00964 (2019)

[7] Wang, Y., Zhao, Y., Callcut, R., Petzold, L.: Integrating physiological time series and clinical notes with transformer for early prediction of sepsis. arXiv preprint arXiv:2203.14469 (2022)

[8] Luo, R., Gong, M., Li, C.: Pt3: A transformer-based model for sepsis death risk prediction via vital signs time series. In: 2023 International Joint Conference on Neural Networks (IJCNN), pp. 1–9 (2023). IEEE

[9] Duffy, G., Cheng, P.P., Yuan, N., He, B., Kwan, A.C., Shun-Shin, M.J., Alexander, K.M., Ebinger, J., Lungren, M.P., Rader, F., *et al.*: High-throughput precision phenotyping of left ventricular hypertrophy with cardiovascular deep learning. JAMA cardiology **7**(4), 386–395 (2022)

[10] Baytas, I.M., Xiao, C., Zhang, X., Wang, F., Jain, A.K., Zhou, J.: Patient subtyping via time-aware lstm networks. In: Proceedings of the 23rd ACM SIGKDD International Conference on Knowledge Discovery and Data Mining, pp. 65–74 (2017)

[11] Choi, E., Bahadori, M.T., Sun, J., Kulas, J., Schuetz, A., Stewart, W.: Retain: An interpretable predictive model for healthcare using reverse time attention mechanism. Advances in ne

RETRACTED ARTICLE

[12] Wang, Q., Chen, G., Jin, X., Ren, S., Wang, G., Cao, L., Xia, Y.: Bit-mac: Mortality prediction by bidirectional time and multi-feature attention coupled network on multivariate irregular time series. Computers in Biology and Medicine **155**, 106586 (2023)

[13] Wang, Y., Huang, N., Li, T., Yan, Y., Zhang, X.: Medformer: A multi-granularity patching transformer for medical time-series classification. arXiv preprint arXiv:2405.19363 (2024)

[14] Nie, Y., Nguyen, N.H., Sinthong, P., Kalagnanam, J.: A time series is worth 64 words: Long-term forecasting with transformers. arXiv preprint arXiv:2211.14730 (2022)

[15] Wu, Z., Pan, S., Long, G., Jiang, J., Chang, X., Zhang, C.: Connecting the dots: Multivariate time series forecasting with graph neural networks. In: Proceedings of the 26th ACM SIGKDD International Conference on Knowledge Discovery & Data Mining, pp. 753–763 (2020)

[16] Bai, L., Yao, L., Li, C., Wang, X., Wang, C.: Adaptive graph convolutional recurrent network for traffic forecasting. Advances in neural information processing systems **33**, 17804–17815 (2020)

[17] Xu, H., Duan, Z., Wang, Y., Feng, J., Chen, R., Zhang, Q., Xu, Z.: Graph partitioning and graph neural network based hierarchical graph matching for graph similarity computation. Neurocomputing **439**, 348–362 (2021)

[18] Kipf, T.N., Welling, M.: Semi-supervised classification with graph convolutional networks. arXiv preprint arXiv:1609.02907 (2016)

[19] Veličković, P., Cucurull, G., Casanova, A., Romero, A., Lio, P., Bengio, Y.: Graph attention networks. arXiv preprint arXiv:1710.10903 (2017)

[20] Xu, D., Ruan, C., Korpeoglu, E., Kumar, S., Achan, K.: Inductive representation learning on temporal graphs. arXiv preprint arXiv:2002.07962 (2020)

[21] Hamilton, W., Ying, Z., Leskovec, J.: Inductive representation learning on large graphs. Advances in neural information processing systems **30** (2017)

[22] Li, M., Zhu, Z.: Spatial-temporal fusion graph neural networks for traffic flow forecasting. In: Proceedings of the AAAI Conference on Artificial Intelligence, vol. 35, pp. 4189–4196 (2021)

[23] Wang, S., Liu, J.: Tagnet: Temporal aware graph convolution network for clinical information extraction. In: 2020 IEEE International Conference on Bioinformatics and Biomedicine (BIBM), pp. 2105–2108 (2020). IEEE

RETRACTED ARTICLE

[24] Zha, D., Lai, K.-H., Zhou, K., Hu, X.: Towards similarity-aware time-series classification. In: Proceedings of the 2022 SIAM International Conference on Data Mining (SDM), pp. 199–207 (2022). SIAM

[25] Bogaerts, T., Masegosa, A.D., Angarita-Zapata, J.S., Onieva, E., Hellinckx, P.: A graph cnn-lstm neural network for short and long-term traffic forecasting based on trajectory data. Transportation Research Part C: Emerging Technologies **112**, 62–77 (2020)

[26] Zhou, J., Cui, G., Hu, S., Zhang, Z., Yang, C., Liu, Z., Wang, L., Li, C., Sun, M.: Graph neural networks: A review of methods and applications. AI open **1**, 57–81 (2020)

[27] Huang, Q., Jiang, J., Rao, X.S., Zhang, C., Han, Z., Zhang, Z., Wang, X., He, Y., Xu, Q., Zhao, Y., et al.: Benchtemp: A general benchmark for evaluating temporal graph neural networks. arXiv preprint arXiv:2308.16385 (2023)

[28] Candel, B.G., Duijzer, R., Gaakeer, M.I., Ter Avest, E., Sir, Ö., Lameijer, H., Hessels, R., Reijnen, R., Zwet, E.W., Jonge, E., *et al.*: The association between vital signs and clinical outcomes in emergency department patients of different age categories. Emergency Medicine Journal **39**(12), 903–911 (2022)

[29] Damluji, A.A., Van Diepen, S., Katz, J.N., Menon, V., Tamis-Holland, J.E., Bakitas, M., Cohen, M.G., Balsam, L.B., Chikwe, J., Arteriosclerosis, T., Cardiovascular Surgery, V.B.C., Anesthesia;, Cardiovascular, C., Nursing, S.: Mechanical complications of acute myocardial infarction: a scientific statement from the american heart association. Circulation **144**(2), 16–35 (2021)

[30] Huang, Y.-L., Hu, Z.-D.: Lower mean corpuscular hemoglobin concentration is associated with poorer outcomes in intensive care unit admitted patients with acute myocardial infarction. Annals of translational medicine **4**(10) (2016)

[31] Ronco, C., Haapio, M., House, A.A., Anavekar, N., Bellomo, R.: Cardiorenal syndrome. Journal of the American College of Cardiology **52**(19), 1527–1539 (2008) https://doi.org/10.1016/j.jacc.2008.07.051

[32] Zhang, Y.-J., *et al.*: Cross-modal contrastive learning for abnormality detection and localization in chest x-rays with radiomics using a feedback loop. Nature Biomedical Engineering **7**, 1456–1469 (2023)

[33] Tipirneni, S., Reddy, C.K.: Self-supervised transformer for sparse and irregularly sampled multivariate clinical time-series. In: Conference on Health, Inference, and Learning, pp. 199–213 (2022)

[34] Shickel, B., *et al.*: Deep ehr: a survey of recent advances in deep learning techniques for electronic health record analysis. IEEE Journal of Biomedical and Health Informatics **22**(5), 1589–1604 (2018)

RETRACTED ARTICLE

[35] Rocheteau, E., Liò, P., Hyland, S.: Temporal pointwise convolutional networks for length of stay prediction in the intensive care unit. In: Proceedings of the Conference on Health, Inference, and Learning, pp. 58–68 (2021)

[36] Horn, M., Moor, M., Bock, C., Rieck, B., Borgwardt, K.: Set functions for time series. In: International Conference on Machine Learning, pp. 4353–4363 (2020). PMLR

[37] Evans, L., *et al.*: Surviving sepsis campaign: international guidelines for management of sepsis and septic shock 2021. Critical Care Medicine **49**(11), 1063–1143 (2021)

[38] Oude Lansink-Hartgring, A., *et al.*: Dysnatremia in the icu: prevalence, clinical correlates, and outcomes. Critical Care **27**, 89 (2023)

[39] Ostermann, M., *et al.*: Acute kidney injury. Nature Reviews Nephrology **19**, 401–417 (2023)

[40] Topol, E.J.: High-performance medicine: the convergence of human and artificial intelligence. Nature Medicine **25**(1), 44–56 (2019)

[41] Ghassemi, M., *et al.*: False hope in clinical ai: empirical analysis of phase 3 trials. The Lancet Digital Health **3**(10), 605–615 (2021)

[42] Ginsburg, G.S., Phillips, K.A.: Precision medicine: from science to value. Health affairs **37**(5), 694–701 (2018)

[43] Kelly, C.J., Karthikesalingam, A., Suleyman, M., Corrado, G., King, D.: Key challenges for delivering clinical impact with artificial intelligence. BMC medicine **17**(1), 195 (2019)

[44] Pollard, T.J., Johnson, A.E., Raffa, J.D., Celi, L.A., Mark, R.G., Badawi, O.: The eicu collaborative research database, a freely available multi-center database for critical care research. Scientific data **5**(1), 1–13 (2018)

[45] He, K., Zhang, X., Ren, S., Sun, J.: Deep residual learning for image recognition. In: Proceedings of the IEEE Conference on Computer Vision and Pattern Recognition, pp. 770–778 (2016)

[46] Mataraso, S.J., Espinosa, C.A., Seong, D., Reincke, S.M., Berson, E., Reiss, J.D., Kim, Y., Ghanem, M., Shu, C.-H., James, T., et al.: A machine learning approach to leveraging electronic health records for enhanced omics analysis. Nature Machine Intelligence, 1–14 (2025)

[47] Prosperi, M., Ghosh, S., Chen, Z., Salemi, M., Lyu, T., Zhao, J., Bian, J.: Causal ai with real world data: Do statins protect from alzheimer's disease onset? In: Proceedings of the 5th International Conference on Medical and Health Informatics,

RETRACTED ARTICLE

pp. 296–303 (2021)

[48] Lipton, Z.C., Kale, D.C., Wetzel, R., *et al.*: Modeling missing data in clinical time series with rnns. Machine Learning for Healthcare **56**(56), 253–270 (2016)

[49] Obermeyer, Z., *et al.*: Dissecting racial bias in an algorithm used to manage the health of populations. Science **366**(6464), 447–453 (2019)

[50] Beaulieu-Jones, B.K., *et al.*: Trends and disparities in model performance over time. Nature Medicine **27**, 2248–2256 (2021)

[51] Kaissis, G.A., *et al.*: Secure, privacy-preserving and federated machine learning in medical imaging. Nature Machine Intelligence **2**, 305–311 (2020)

[52] Johnson, A.E., Pollard, T.J., Shen, L., Lehman, L.-w.H., Feng, M., Ghassemi, M., Moody, B., Szolovits, P., Anthony Celi, L., Mark, R.G.: Mimic-iii, a freely accessible critical care database. Scientific data **3**(1), 1–9 (2016)

[53] Wang, S., McDermott, M.B., Chauhan, G., Ghassemi, M., Hughes, M.C., Naumann, T.: Mimic-extract: A data extraction, preprocessing, and representation pipeline for mimic-iii. In: Proceedings of the ACM Conference on Health, Inference, and Learning, pp. 222–235 (2020)

[54] Elbadawi, A., Elgendy, I.Y., Mahmoud, K., Barakat, A.F., Mentias, A., Mohamed, A.H., Ogunbayo, G.O., Megaly, M., Saad, M., Omer, M.A., *et al.*: Temporal trends and outcomes of mechanical complications in patients with acute myocardial infarction. JACC: Cardiovascular Interventions **12**(18), 1825–1836 (2019)

[55] Hyland, S.L., Faltys, M., Hüser, M., Lyu, X., Gumbsch, T., Esteban, C., Bock, C., Horn, M., Moor, M., Rieck, B., *et al.*: Early prediction of circulatory failure in the intensive care unit using machine learning. Nature medicine **26**(3), 364–373 (2020)

[56] Yèche, H., Kuznetsova, R., Zimmermann, M., Hüser, M., Lyu, X., Faltys, M., Rätsch, G.: Hirid-icu-benchmark–a comprehensive machine learning benchmark on high-resolution icu data. arXiv preprint arXiv:2111.08536 (2021)

[57] Wornow, M., Thapa, R., Steinberg, E., Fries, J., Shah, N.: Ehrshot: An ehr benchmark for few-shot evaluation of foundation models. Advances in Neural Information Processing Systems **36** (2024)

[58] Xu, K., Hu, W., Leskovec, J., Jegelka, S.: How powerful are graph neural networks? arXiv preprint arXiv:1810.00826 (2018)

RETRACTED ARTICLE

Acknowledgements. MM is supported by the Rhodes Trust and the EPSRC CDT Health Data Science. TZ is supported by the Royal Academy of Engineering. We appreciate the support of Max Buhlan with Figure 1.

Author contributions. M.M., S.M., and T.Z. conceived and designed the study. M.M. and S.M. performed statistical and machine learning analyses. P.W. and T.Z. provided administrative, technical, and material support. M.M. and S.M. drafted the manuscript. P.W. and T.Z. supervised the study. All authors aided in the interpretation of data, and critical revision of the manuscript. M.M., S.M., and T.Z. had access to and verified all of the data in the study.

Competing interests. We declare no competing interests.

Supplementary information. Supplementary material is contained in a separate file.

RETRACTED ARTICLE
